# Inefficient and unique processing of social–emotional interference in school-aged children with high-functioning autism spectrum disorder

**DOI:** 10.3389/fpsyt.2024.1412533

**Published:** 2024-10-09

**Authors:** Qing-Xin Chen, Qi Chen, Kun Zhai, Hui-Ting Chen, Yu-Lan Wu, Jin-Ming Liu, Yu Jin

**Affiliations:** ^1^ Department of Maternal and Child Health, School of Public Health, Sun Yat-sen University, Guangzhou, China; ^2^ Department of Children's Healthcare, Beijing University of Chinese Medicine Shenzhen Hospital (Longgang), Shenzhen, China; ^3^ The First Affiliated Hospital, Sun Yat-sen University, Guangzhou, China

**Keywords:** autism spectrum disorder, interference control, emotional face, flanker, cognitive strategies, children, information load

## Abstract

**Introduction:**

Interest is growing in investigating the ability of children with autism spectrum disorder (ASD) to process social information under conflicting and complex environments. However, few studies have employed objective behavioral measures to directly explore the underlying profile of social–emotional interference control.

**Methods:**

In the current study, 53 children with ASD and 53 typically developing (TD) control, aged 6–12 years, completed a set of modified flanker tasks involving arrows, schematic faces, same real faces (with facial interference by the same person), and different real faces (with facial interference by different people), respectively. Response time in incongruent (RT^Inc^) and congruent conditions (RT^Con^), percentage of errors in incongruent (%Error^Inc^) and congruent conditions (%Error^Con^), and flanker effect calculated by ΔRT = (RT^Inc^ − RT^Con^)/RT^Con^ and Δ%Error = %Error^Inc^ − %Error^Con^ were used as outcome metrics.

**Results:**

We obtained three major results: (1) the ASD group had longer RT^Inc^ and RT^Con^ compared to the TD group in the arrow, schematic-face, and same real-face tasks; (2) compared with the performance in the arrow flanker task, both groups exhibited longer RTs and reduced ΔRTs in the same real-face task; however, in the schematic-face task, longer RT and reduced ΔRT were exhibited in the TD group, but not in the ASD group; and (3) in the different real-face task, ASD group had higher %Error than the TD group, and %Error was negatively correlated with RT only in the ASD group.

**Conclusion:**

The current study delineates the inefficient processing of social–emotional interference in school-aged children with ASD and further suggests that these children might adopt a relatively optimized strategy like symbolization when dealing with emotional conflict. However, such compensatory cognitive strategies may be exhausted along with the increase in information load. These findings provide new perspectives of considering the difference more than difficulty in the cognitive profile of ASD, which will benefit the development of targeted behavioral interventions.

## 1 Introduction

Autism spectrum disorder (ASD) is a prevalent neurodevelopmental condition that presents a wide range of challenges in social interaction, communication, and behavior (1–3). Abnormal social behaviors are often paralleled by atypical or inefficient cognitive processing of social information (4–6). Social contexts are filled with complex and conflicting information, requiring individuals to select and control (7). Interference control, which refers to the ability to monitor and resist irrelevant information in order to give appropriate responses (8–12), is critical for efficient functioning in daily life (7). Numerous studies have reported the deficiency of interference control among individuals with ASD, e.g., they presented longer response time (13) and/or higher response error rate (14–17) than typically developing (TD) controls in the Eriksen arrow or letter flanker tasks (18). However, when extending this perspective to interference control involving social information, the specific profile in children with ASD remains unclear (19).

As the most representative carrier of social information, emotional faces convey rich social clues and shape the way we interact in the social environment (20, 21). Although a growing body of evidence recently has suggested that children with ASD without intellectual disability were able to distinguish basic facial emotion and identity (22–25), they would still be stuck in great challenges under conflict conditions (26, 27). In addition to the studies reporting the deficiency of nonsocial interference control among children with ASD, as reviewed above, a recent research also found that children with ASD make more errors than TD children when processing facial information under the influence of interfering stimuli (28). Dickter et al. (29) found that participants with higher autistic traits responded more slowly in the emotional face flanker task compared to those with lower autistic traits. Given this, it is reasonable to speculate that children with ASD may have difficulties with interference control involving emotional faces. Therefore, by combining emotional face stimuli with the flanker paradigm, the current study aims to directly examine the profile of social–emotional interference control in children with ASD.

Significantly, the emotional face per se can influence cognitive processing (30, 31), but this effects differs between ASD and TD individuals, which likely stems from their diverse cognitive patterns (32). Studies on TD individuals suggest that emotional faces may cause less interference compared to stimuli used in the traditional flanker tasks (arrows or letters) because of their perceptual specialty or complexity (21, 33, 34). On the contrary, children with ASD may possess detail-strengthened characteristics in visual processing (35–37) and tend to ignore social clues and focus more on areas where physical properties are highlighted (38–40). Therefore, it is suspected that individuals with ASD might make more efforts to detect the differences in facial details instead of perceiving emotional expressions. Here, we introduced the schematic face, which carries both social and symbolic attributes, to dissociate the emotional and perceptual effects from the behavioral aspect (41–43).

Another important consideration in facial flanker tasks is that the same face presentation could not accurately mirror real-life situations where individuals frequently engage with multiple people simultaneously (33). Additionally, previous studies have noted that children may focus on small visual changes to determine emotional valence when the target and flanker stimuli are from the same individual (21, 44). The relevant factor affecting cognitive control is information load (i.e., information complexity) (34, 45–47). Specifically, TD individuals presented prolonged response times, increased error rates, and reduced flanker effects (the degree of increase in response time or error rate due to the incongruent interfering stimuli) as information load increases (33, 47). However, it remains an open question whether and how information load would influence the profile of social–emotional interference control in children with ASD. Based on this, the current study further designed three types of emotional face flanker tasks: schematic face, real face with the same face interference, and real face with different face interference.

The purpose of the current study was to examine social–emotional interference control in children with ASD using objective behavioral measures, explore potential cognitive strategies, and further explore the influence of information load. Drawing on the literature concerning social interference control and the visual processing style in ASD, we hypothesized that (1) social–emotional interference control in children with ASD was inefficient or impaired, which may be presented by longer response times or higher error rates than TDs in the emotional face flanker tasks; (2) children with ASD may adopt detail-focused strategies in social–emotional interference control, potentially reflected in similar performance between the arrow and schematic-face flanker tasks; and (3) the increasing complexity of emotional faces may disrupt the cognitive processing of children with ASD during interference control, potentially resulting in more errors than TD in the real-face flanker tasks.

## 2 Materials and methods

The study was approved by the Medical Ethics Committee of Sun Yat-sen University (2019-No.090). Informed consent was obtained from all participants’ guardians before the onset of the experiment.

### 2.1 Participants

A total of 111 children (including 56 children with ASD and 55 TD children) aged 6–12 years were recruited between October 2020 and October 2021 at the Research Center for Child and Adolescent Psychology, Behavior and Development at Sun Yat-Sen University.

All participants with ASD were required to have a historical diagnosis confirmed by a combination of the Childhood Autism Rating Scale (CARS) and an expert clinician at a tertiary hospital. Two professional child psychiatrists reconfirmed all diagnoses according to the *Diagnostic and Statistical Manual of Mental Disorders, Fifth Edition* (DSM-5) criteria (1). The Chinese version of the Wechsler Intelligence Scale for Children, Fourth Edition (C-WISC-IV) (48) was used to measure the intelligence quotient (IQ) of all participants. Social Responsiveness Scale (SRS) (49) was used to quantify autistic traits. All included children were required to have a full-scale intelligence quotient (FSIQ) score > 80 (46, 50) and normal naked vision or corrected vision in both eyes.

Children in both groups were excluded if they (1) had a severe physical illness or other neurological or psychiatric disorders except for ASD, including learning disability (LD) and attention deficit hyperactivity disorder (ADHD); (2) had used psychoactive drugs within the past 5 weeks; (3) had difficulty pressing buttons easily during the practice phase; (4) could not understand or complete all tests; or (5) had an error rate exceeding 50% in the arrow flanker task (congruent condition) or a total omission rate of more than three times the standard deviations (SDs). Additionally, children in the TD group were excluded if they had high autistic traits (SRS total score > 75) (51).

### 2.2 Apparatus

The stimuli were presented on a 15-in. color monitor (1,920 × 1,080 pixel resolution). The subjects’ eyes were positioned at the center of the screen, maintaining a horizontal distance of 40 cm, as required. Presentation of stimuli, timing operations, and data collection were controlled by a computer using E-Prime 2.0 software. Responses were collected using a standard computer keyboard.

### 2.3 Flanker paradigm

**Figure 1:**
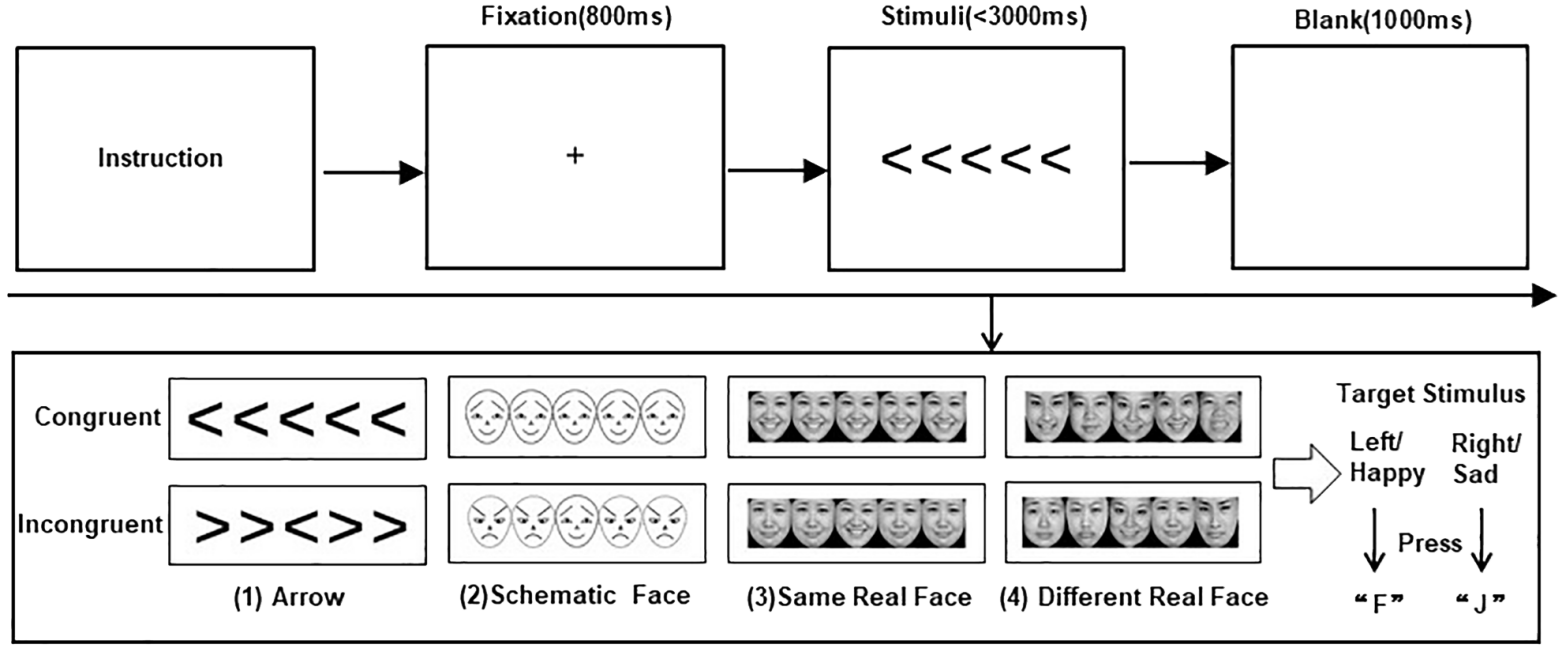
Stimuli and trial sequences. The top of the figure shows the trial sequences for the four flanker tasks. The bottom of the figure presents the stimuli for (1) arrows, (2) schematic faces, (3) same real faces, and (4) different real faces, each in congruent and incongruent conditions, respectively.

Stimuli and trial sequences are shown in **Figure 1**.

In the current study, we used four flanker tasks of arrow and emotional faces to explore the impact of congruent and incongruent nonsocial and social–emotional interference information on target stimuli recognition in children with ASD. First, participants were requested to complete the classic arrow flanker task, where the central target arrow was flanked by four interference arrows, with adjacent stimuli separated by 5° of visual angle. Participants were asked to press a button to indicate the direction of the target arrow (the “F” button for the left and the “J” button for the right) while disregarding the interference arrows. A congruent condition is defined as a condition where the direction of the interference arrow is consistent with that of the target arrow; otherwise, it is an incongruent condition.

Participants were then required to complete three face flanker tasks, including schematic-face, same real-face, and different real-face tasks. They were asked to identify the central target facial emotion by pressing a button (F button for happy and J for sad). In the schematic-face flanker task, positive and negative emotional faces had identical physical components but were in opposite directions, as described by Ohman et al. (52). Photographs of real faces (including happy and sad expressions) were all from the Chinese Facial Affective Picture System (CFAPS) (53). The emotional intensity of these real-face materials was between 3.5 and 8.5, with an identification degree greater than 80%. In the same real-face flanker task, the photographs of the same person were presented as target and interference stimuli. To prevent participants from using specific facial features for recognition, the same real-face task used photographs of 10 adults (five women and five men), each providing one happy face and one sad face. In the different real-face flanker task, the photographs of five different people were presented as target and interference stimuli, where pictures of a total of 50 adults (25 women and 25 meb) were used.

### 2.4 Procedure

Each flanker task consisted of a practice block (eight trials) and three formal blocks (a total of 60 trials). Stimuli were randomly presented within each block, with congruent and incongruent conditions each occurring with a 50% probability. Before each task began, the instruction was patiently explained by a well-trained researcher until the participants fully understood. In each trial, a fixation “+” of 800 ms was first presented in the center of the screen, and participants were requested to fix their eyes on the “+” to ensure stable binocular convergence. Subsequently, the stimuli were randomly presented for not more than 3,000 ms until a response was detected, and participants were asked to respond as quickly and accurately as possible. A blank screen of 1,000 ms (stimulation interval) automatically appeared after a response or after 3,000 ms, before moving on to the next trial. The program recorded whether a button was pressed, the response results, and the response time.

In the practice block, no interfering stimuli were presented on either side of the target stimulus, and visual feedback of “correct” (blue) or “wrong” (red) was provided at the end of each trial. Only participants with more than 60% accuracy in the practice block could enter the formal test; otherwise, they would re-enter the practice block. All participants were allowed to practice no more than three times in each flanker task and could rest for 2 min after the practice block and between tasks.

### 2.5 Data analysis

Corrected response time (RT) and percentage of error (%Error) were used to represent the characteristics of interference control, including RT in incongruent condition (RT^Inc^), RT in congruent condition (RT^Con^), %Error in incongruent condition (%Error^Inc^), and %Error in congruent condition (%Error^Con^). The flanker effect was calculated as follows: (1) ΔRT = (RT^Inc^ − RT^Con^)/RT^Con^, indicating the extent of RT extension due to the incongruent interfering stimuli (9); (2) Δ%Error = %Error^Inc^ − %Error^Con^, indicating the degree of increase in %Error due to the incongruent interfering stimuli.

Data analysis was carried out using SPSS, Version 25.0 (SPSS Inc., Chicago, IL, USA). Initially, we conducted Kolmogorov–Smirnov tests to inspect the distribution of quantitative variables (see **Supplementary Table SR1**). Based on the results, parametric tests were employed for normally distributed variables, while nonparametric tests were used for nonnormally distributed variables. All statistical analyses of the behavioral tasks were conducted in two parts.

The first part of the study compared the characteristics of interference control in nonsocial and social–emotional information. A generalized linear mixed model (GLMM) with 3 stimulus types (arrow, schematic face, and same real face) × 2 conflict conditions (congruent condition and incongruent condition) × 2 groups (ASD group and TD group) was used, with RT and %Error set as dependent variables. Stimulus type, conflict condition, group, and their interactions were taken as fixed effects, with subjects as random effects. *Post-hoc* tests included the following: (1) the effect of group was analyzed using independent sample *t*-test or rank sum tests; (2) the effect of conflict condition was tested using paired sample *t*-tests and rank sum tests; and (3) the effect of stimulus type was compared using the Bonferroni method, adjusting the test level *α* to 0.025 for multiple comparisons. Specifically, the performance of social flanker tasks (schematic face and same real face) was compared with that of the nonsocial flanker tasks (arrow).

The second part of the study compared the characteristics of interference control for emotional faces under different information loads. A GLMM was employed with 3 stimulus types (schematic face, same real face, and different real face) × 2 conflict conditions (congruent and incongruent condition) × 2 groups (ASD group and TD group) was used. The *post-hoc* tests were the same as those in the first part. Specifically, as for the effect of stimulus type, the characteristics of social–emotional interference control between the two adjacent groups (schematic face and same real face; same real face and different real face) were compared. The test level *α* was adjusted to 0.025 to correct for multiple comparisons. Additionally, Spearman correlational analyses were conducted for RT and %Error to determine whether a speed-accuracy trade-off [defined as increased %Error accompanied by decreased RT (46)] was present in the ASD group during the different real-face tasks. The size of the test was set at *α* = 0.05.

## 3 Results

### 3.1 Demographic characteristics

Three children with ASD were excluded because they could not complete the behavioral tasks required, and two participants in the TD group were excluded due to high autistic traits (SRS total score > 75). As a result, the final sample used for data analysis included 53 children with ASD (M = 8.69 years, SD = 1.88) and 53 TD children (M = 9.03 years, SD = 1.48). The demographic characteristics of the ASD and TD groups are shown in **Table 1**. There were no significant differences in age, gender, and IQ between the two groups (*p* > 0.05). Children with ASD showed significantly higher total and subscale scores in SRS compared to TD children (*p* < 0.001).

**Table 1:** Demographic characteristics of the ASD and TD groups.

Participant characteristics	ASD (*n* = 53)	TD (*n* = 53)	*t/χ* ^2^	*p-value* ^a^
	Mean ± SD/*N* (%)	Mean ± SD/*N* (%)		
**Age (years)**	8.69 ± 1.88	9.03 ± 1.48	− 1.015	0.312
**Sex**				
Male	46 (86.79)	39 (73.58)	2.910	0.088
Female	7 (13.21)	14 (26.42)		
**FSIQ**	104.68 ± 14.76	108.87 ± 12.48	− 1.577	0.118
Verbal comprehensive index	107.64 ± 18.37	113.02 ± 16.92	− 1.567	0.120
Perceptual reasoning index	109.02 ± 14.72	109.66 ± 11.70	− 0.248	0.804
Working memory index	100.58 ± 13.72	101.81 ± 11.97	− 0.490	0.625
Processing speed index	95.70 ± 17.54	99.55 ± 11.24	− 1.345	0.182
**SRS total score**	74.49 ± 20.60	39.53 ± 13.93	10.233	< 0.001
Social awareness	10.40 ± 2.58	7.72 ± 2.68	5.244	< 0.001
Social cognition	14.57 ± 4.91	8.34 ± 3.91	7.225	< 0.001
Social communication	24.58 ± 7.46	10.77 ± 4.67	11.424	< 0.001
Social motivation	11.55 ± 4.75	7.19 ± 4.17	5.025	< 0.001
Restricted and repetitive behaviors	13.58 ± 5.35	5.51 ± 3.91	8.878	< 0.001

ASD, autism spectrum disorder; TD, typically developing; SD, standard deviation; N (%), numbers (percent); FSIQ, full-scale intelligence quotient; SRS, Social Responsiveness Scale.

a The *p*-value for the independent sample *t*-test or Chi-square between the two groups.

Given the good comparability of the demographic characteristics between the ASD and TD groups, comparisons between the two groups did not require adjustments for any of the variables. Therefore, the group effect presented unadjusted results. Additionally, since RT was negatively correlated with age and full-scale intelligence quotient (FSIQ) among children with ASD (see **Supplementary Table SR3**), the analysis of RT considering age and FSIQ (tested by analysis of covariance) will be provided in the **Supplementary Materials**.

### 3.2 Characteristics of interference control on social–emotional and nonsocial flanker tasks

The 3 × 2 × 2 GLMM was conducted to analyze the characteristics of interference control on nonsocial and social–emotional information. When RT was set as the dependent variable, all main effects and all interaction effects were significant: conflict condition (*F* = 146.577, *p* < 0.001), group (*F* = 16.635, *p* < 0.001), stimulus type (*F* = 104.284, *p* < 0.001), conflict condition × group (*F* = 6.505, *p* = 0.011), stimulus type × group (*F* = 7.631, *p* = 0.001), and stimulus type × conflict condition (*F* = 4.710, *p* = 0.009). When %Error was set as the dependent variable, no significant interaction effects or main effects related to the group were found. Thus, all subsequent analyses in this part focused on the differences in RT.

**Figure 2:**
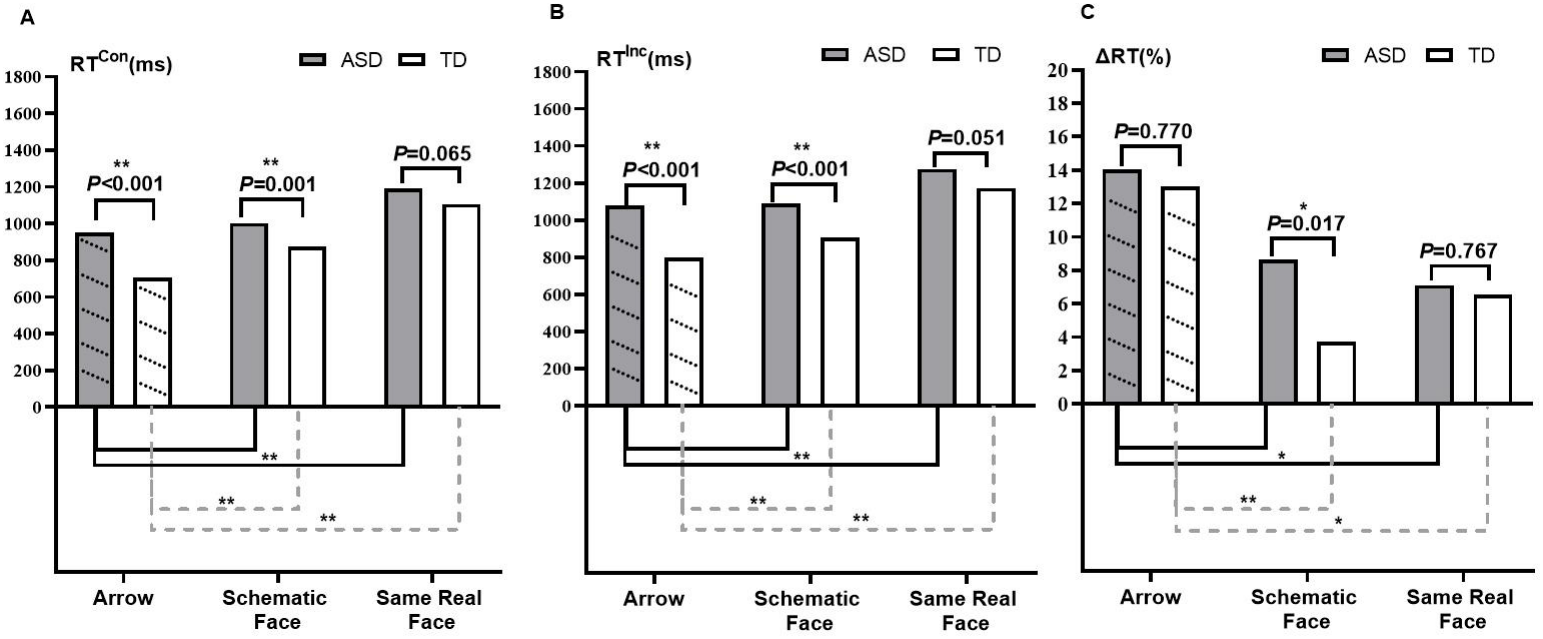
Characteristics of interference control on social–emotional and nonsocial flanker tasks among ASD and TD groups. Note: Abbreviations: ASD, autism spectrum disorder; TD, typically developing; RT, respond time, RT^Con^, RT in congruent condition; RT^Inc^, RT in incongruent condition; ΔRT, flanker effect of RT (ΔRT = (RT^Inc^ − RT^Con^)/RT^Con^). Results of group differences in RT^Con^ **(A)**, RT^Inc^ **(B)**, and ΔRT **(C)** are shown above the bar chart, tested by independent sample *t*-tests. ^*^ *p* < 0.05; ^**^ *p* < 0.01. Results of the comparison of the social–emotional flanker task (schematic face and real face) with the nonsocial flanker task (arrow) are shown under the bar chart, tested by the Bonferroni method. Multiple comparisons are corrected. ^*^ *p* < 0.025; ^**^ *p* < 0.005.

**Table 2** presents the simple effect of conflict conditions. RT^Inc^ was longer than RT^Con^ in the three tasks—arrow, schematic face, and same real face—in both ASD and TD groups (all *p* < 0.05). It is evident that both groups showed flanker effects in these tasks, as their ΔRTs were not equal to zero. Results of the simple effect of group (**Figures 2A, B**) revealed significant differences of both RT^Con^ and RT^Inc^ in arrow and schematic-face tasks between ASD and TD groups (*p* < 0.05), and the difference of RT^Con^ (*t* = 1.864, *p* = 0.065) and RT^Inc^ (*t* = 1. 973, *p* = 0.051) in the same real-face task were marginally significant between the two groups. These results remained stable after controlling for children’s age and FSIQ (**Supplementary Table S1**). Specifically, the ASD group had longer RT^Con^ and RT^Inc^ compared to the TD group in the arrow, schematic-face, and same real-face tasks. As for ΔRT, group differences were significant in the schematic-face task (*t* = 2.416, *p* = 0.017) instead of the arrow (*t*=0.294, *P*=0.770) and the same real-face tasks (*t* = 0.297, *p* = 0.767). In particular, the ASD group had a larger ΔRT than the TD group in the schematic-face task (**Figure 2C**).

**Table 2:** Characteristics of interference control in four flanker tasks among ASD and TD groups.

	Stimulus types	ASD (*n* = 53)				TD (*n* = 53)			
		Mean ± SD/median (Q1, Q3)				Mean ± SD/median (Q1, Q3)			
		Congruent condition	Incongruent condition	*t/z*	*p-value*	Congruent condition	Incongruent condition	*t/z*	*p-value*
**RT (ms)** ^a^	**Arrow**	950.18 ± 334.29	1,078.07 ± 386.26	− 6.255	**< 0.001**	704.66 ± 174.77	799.89 ± 257.21	− 4.963	**< 0.001**
	**Schematic face**	1,002.65 ± 211.41	1,090.29 ± 259.88	− 5.420	**< 0.001**	873.75 ± 192.73	905.96 ± 207.75	− 3.068	**0.003**
	**Same real face**	1,192.85 ± 255.94	1,275.63 ± 298.03	− 4.128	**< 0.001**	1,106.95 ± 216.79	1,174.02 ± 227.52	− 4.807	**< 0.001**
	**Different real face**	1,240.13 ± 329.96	1,205.92 ± 313.29	1.844	0.071	1,148.46 ± 238.40	1,161.58 ± 251.95	− 0.819	0.417
**%Error(%)** ^b^	**Arrow**	0 (0, 3.33)	6.67 (0, 24.14)	− 4.735	**< 0.001**	0 (0, 6.67)	6.67 (3.33, 13.79)	− 3.668	**< 0.001**
	**Schematic face**	3.33 (0, 10.00)	6.67 (0, 7.14)	< 0.001	1.000	6.67 (3.33, 10.00)	6.90 (3.33, 16.67)	− 2.644	**0.008**
	**Same real face**	6.90 (3.33, 16.67)	10.34 (6.67, 20.00)	− 2.428	**0.015**	10.00 (3.33, 10.34)	10.00 (6.67, 13.79)	− 1.833	0.067
	**Different real face**	20.69 (13.33, 29.63)	16.67 (10.71, 23.33)	2.124	**0.034**	13.33 (10.00, 16.67)	10.34 (6.67, 16.67)	1.478	0.140

The values marked in bold mean *p* < 0.05.

ASD, autism spectrum disorder; TD, typically developing; RT, response time; %Error, percentage of error; SD, standard deviation; (Q1, Q3), (first quartile, third quartile).

a Effect of the conflict condition of RT was tested using a paired sample *t*-test.

b Effect of the conflict condition of %Error was tested using a paired sample rank sum test.

Using RT^Con^ and RT^Inc^ from the arrow task as a reference, the results showed differences in social–emotional interference control between the ASD and TD groups. In the ASD group, there were no significant differences between the schematic-face task and the arrow task in either RT^Con^ (*p* = 0.151) or RT^Inc^ (*p* = 0.778). However, both RT^Con^ (*p* < 0.001) and RT^Inc^ (*p* < 0.001) in the same real-face task were longer than those in the arrow task. In the TD group, RT^Con^ (*p* < 0.001) and RT^Inc^ (*p* = 0.002) in the schematic-face task, as well as RT^Con^ (*p* < 0.001) and RT^Inc^ (*p* < 0.001) in the real-face task, were all longer than those in the arrow task (**Figures 2A, B**). Similarly, using ΔRT from the arrow task as a reference, the ASD and TD groups showed divergent flanker effects in the face tasks. In the ASD group, ΔRT in the schematic-face task showed no significant difference (*p* = 0.112), while ΔRT in the real-face task significantly decreased (*p* = 0.014). In the TD group, ΔRT in both the schematic-face (*p* < 0.001) and real-face (*p* = 0.021) tasks significantly decreased (**Figure 2C**).

### 3.3 Characteristics of social–emotional interference control with different information loads

The 3 × 2 × 2 GLMM was conducted to compare the characteristics of interference control across different information loads (schematic face, same real face, and different real face). When RT was set as the dependent variable, interaction effects were significant in terms of stimulus type × group (*F* = 3.096, *p* =0.046), stimulus type × conflict condition (*F* = 14.344, *p* < 0.001), and stimulus type × conflict condition × group (*F* = 5.405, *p* = 0.005). The main effects were significant for the conflict condition (*F* = 37.607, *p* < 0.001), group (*F* = 6.434, *P*=0.011), and stimulus type (*F* = 96.948, *p* < 0.001). When %Error was set as the dependent variable, interaction effects were significant in terms of stimulus type × group (*F* = 15.333, *p* < 0.001) and stimulus type × conflict condition (*F* = 10.822, *p* < 0.001). The main effect was significant for the stimulus type (*F* = 73.124, *p* < 0.001).

Results of the simple effect of group (**Figure 2**; **Table 3**) revealed that, in the schematic-face task, RT^Con^ and RT^Inc^ were longer in the ASD group than in the TD group (*p* < 0.05). In the same real-face task, RT^Con^ and RT^Inc^ showed an increasing trend in the ASD group compared to the TD group (0.05 *< p* < 0.1). In the different real-face task, no significant differences were found in RT^Con^ and RT^Inc^ between the two groups (*p* > 0.05). Additionally, compared with the TD group, the ASD group had a lower %Error^Inc^ (*z* = − 2.245, *p* = 0.025) in the schematic-face task and a higher %Error^Con^ (*z* = 2.314, *p* = 0.021) and %Error^Inc^ (*z* = 2.567, *p* = 0.010) in the different real-face task. Specifically, we found a negative correlation between RT and %Error in the different real-face task for the ASD group (*r_s_* = − 0.378, *p* = 0.005), whereas no such correlation was observed in the TD group (*p* > 0.05).

**Table 3:** Group differences in %Error.

Stimulus types	Conflict condition	ASD (*n* = 53) median (Q1, Q3)	TD (*n* = 53) median (Q1, Q3)	*z*	*p-value* ^a^
**Arrow**	Congruent condition	0 (0, 3.33)	0 (0, 6.67)	− 1.814	0.070
	Incongruent condition	3.51 (0, 13.33)	6.67 (3.33, 16.67)	− 0.760	0.447
**Schematic face**	Congruent condition	3.33 (0, 10.00)	6.67 (3.33, 10.34)	− 1.074	0.283
	Incongruent condition	6.67 (3.33, 10.00)	6.90 (3.33, 16.67)	− 2.245	**0.025**
**Same real face**	Congruent condition	7.02 (3.33, 16.67)	10.00 (3.33, 11.54)	0.404	0.686
	Incongruent condition	10.53 (6.67, 23.33)	10.34 (6.67, 16.67)	0.764	0.445
**Different real face**	Congruent condition	20.34 (13.33, 30.00)	13.79 (10.00, 20.00)	2.314	**0.021**
	Incongruent condition	16.67 (10.71, 26.67)	10.34 (6.67, 20.00)	2.567	**0.010**

Values marked in bold indicate *p* < 0.05. All of *p*-values are unadjusted for any variables.

%Error, percentage of error; ASD, autism spectrum disorder; TD, typically developing; (Q1, Q3), (first quartile, third quartile).

a The *p*-value for the independent sample rank sum test between two groups.

**Figure 3:**
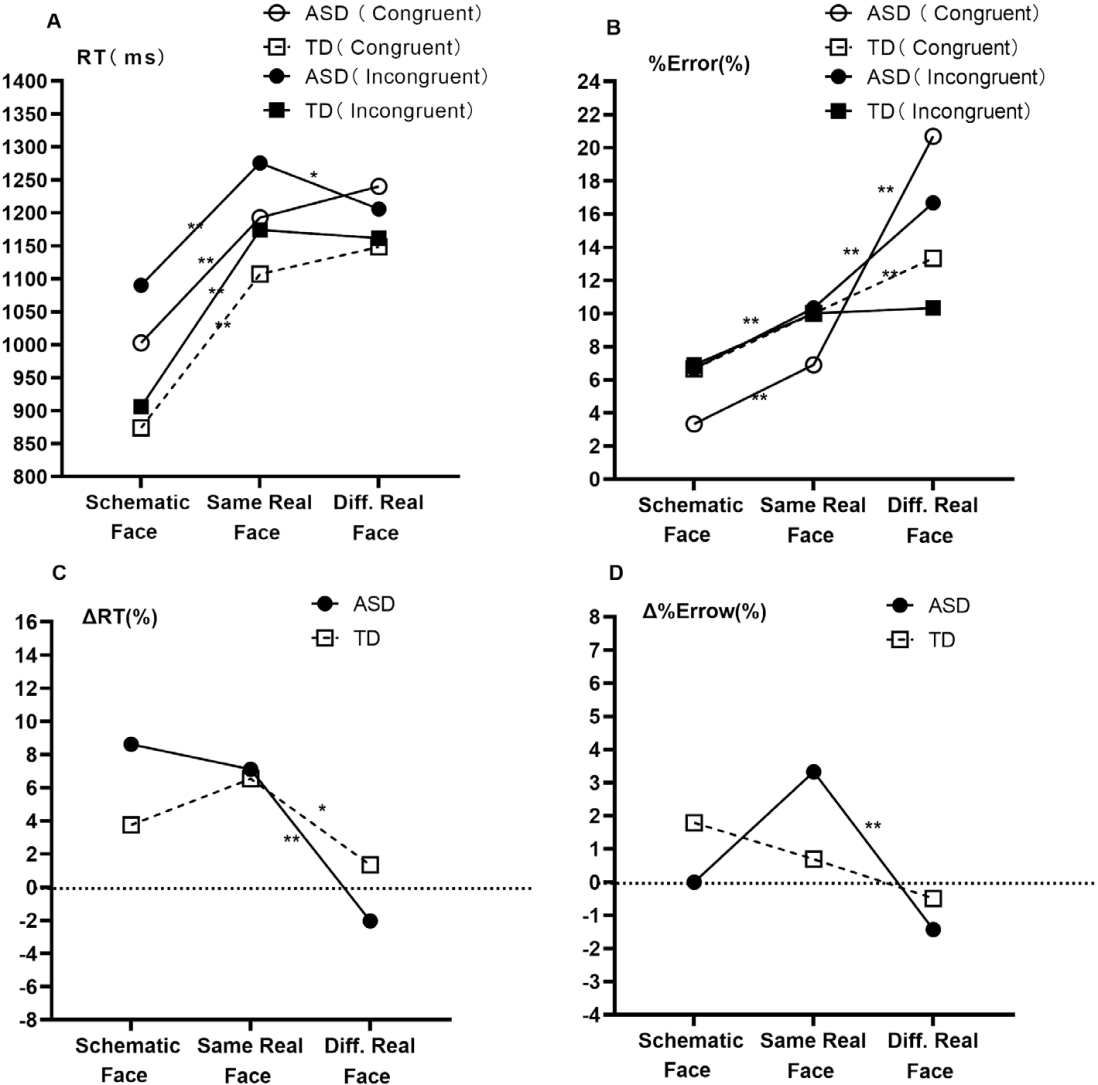
Comparisons of interference control characteristics with different social information loads between ASD and TD groups. Note: Abbreviations: ASD, autism spectrum disorder; TD, typically developing; RT, response time; %Error, percentage of error; ΔRT, flanker effect of RT (ΔRT = (RT^Inc^ − RT^Con^)/RT^Con^); Δ%Error, flanker effect of %Error (Δ%Error = %Error^Inc^ − %Error^Con^); Diff. Real Face, different real face. Means of response time **(A)**, medians of percentage of error **(B)**, means of the flanker effect of response time **(C)**, and medians of the flanker effect of percentage of error **(D)** are shown. Pairwise comparisons of different stimulus types with adjacent information loads are shown and tested using the Bonferroni method. Multiple comparisons are corrected. ^*^ *p* < 0.025; ^**^ *p* < 0.005.

Pairwise comparisons of different stimulus types with adjacent information loads are shown in **Figure 3**. In terms of RT, both the ASD group and the TD group showed prolonged RT^Con^ and RT^Inc^ from the schematic-face task to the same real-face task (*p* < 0.001). However, from the same real-face task to the different real-face task, only the ASD group showed a shorter RT^Inc^ (*p* = 0.008). In terms of %Error, from the schematic-face task to the same real-face task, only the ASD group showed increased %Error^Con^ (*p* = 0.001) and %Error^Inc^ (*p* < 0.001). From the same real-face task to the different real-face task, both groups showed increased %Error^Con^ (*p* < 0.001), while only the ASD group showed increased %Error^Inc^ (*p* < 0.001). Lastly, both groups showed decreased ΔRT and Δ%Error from the same real-face task to the different real-face task, with Δ%Error in the TD group showing marginal significance of (*p* = 0.028, *α* = 0.025).

## 4 Discussion

The current study utilized a set of modified flanker tasks with emotional faces to investigate the ability of social–emotional interference control in school-aged children with ASD, and three key findings were identified. First, children with ASD were inefficient, rather than exactly impaired, in social–emotional interference control. Second, compared with the TD group, children with ASD showed different processing patterns for interference control of emotional faces, which may be related to detail-focused symbolic strategies. Third, the increasing information load of emotional faces further disrupted the interference control in children with ASD, increasing their risk of cognitive crashes.

First of all, the data confirmed that children with ASD presented poorer interference control abilities for both nonsocial and social–emotional information compared to TD children. Specifically, they had longer response times than TD children in both congruent and incongruent conditions in the face and arrow flanker tasks. The results were not only consistent with well-documented findings on processing nonsocial interference (13–17) but also extended the perspectives on the characteristics of social–emotional interference control, which had been studied only to a limited extent before. In a recent study, researchers found that participants with higher autistic traits responded more slowly in the emotional face flanker task than those with lower autistic traits (29). Taken together, these findings suggest that individuals with ASD, affected by nonsocial and social–emotional interference, require more time than TD individuals to process visual information and provide appropriate responses. This delay can strongly impact their ability to function efficiently in daily life and social contexts (7, 14). In addition, the interference stimulus can affect the processing of the target stimulus, no matter the information conveyed by it was congruent or incongruent with that conveyed by the target stimulus. Furthermore, our data demonstrated no group differences in response error rates were found between the ASD and TD groups in the arrow and same real-face tasks. This finding is consistent with studies on participants with high-functioning ASD (9, 54) but contrasts with a previous study that found increased error rates in the arrow flanker task among children with ASD (without estimating verbal IQ) and suggested complete impairment in interference control (14). For the above reason, we believe that the ability to recognize facial emotions under facial interference is relatively intact but inefficient in children with high-functioning ASD (22–25, 55). This raises the probability that the relative intactness of processing facial emotional interference in these children may be attributed to their unique cognitive strategies.

In the current study, we introduced schematic faces as a transition between nonsocial and social stimuli. Taking performance in the arrow flanker task as a reference, the results showed that TD children presented longer response times and a reduced flanker effect in both the schematic-face task and real-face tasks. In contrast, children with ASD performed similarly in the schematic-face task and the arrow flanker task. ASD and TD children were likely to adopt different cognitive patterns when processing the interference of schematic faces, which have dual attributes of sociality and symbolism. Previous studies have pointed out that social information can exacerbate the difficulties of interference control, leading individuals to use more cognitive resources or change their cognitive strategies (56). For TD children, the flanker effect was weaker or sometimes nonexistent in the emotional face flanker tasks (including the schematic-face flanker task) due to the high complexity level, which left little room for automatic processing of emotional facial interference (33, 34). In contrast, the autistic-trait-related cognitive pattern indicates that children with ASD have a better capacity to ignore integral information and identify partial information more easily (8, 36, 57–59). This is similar to findings from eye-tracking tests, which suggest that children with ASD may deconstruct face stimuli into simple symbols, distinguishing emotions such as happiness and sadness based on upturned or downturned mouth corners (25, 60–62). Accordingly, we considered that children with ASD would automatically ignore the social features of schematic faces and alternatively magnify the symbolic features, regarding them as arrow-like symbolic information (25, 63). The symbolic conflicts were easier to detect (33, 56), which may lead to a larger flanker effect in the ASD group than in the TD group, as our data depicted. Moreover, our data also showed that the %Error in the ASD group was unexpectedly lower than that in the TD group in the schematic-face task. We believe this may be related to the reason that children with ASD’s attention preference for physical signs helps them process target stimuli. To some extent, such attention-to-detail strategies could simplify cognitive complexity and enhance their daily functioning. However, the compensation of such cognitive strategies in children with ASD may be limited and ineffectual (64) in more complex, real-face tasks.

Furthermore, we designed threefold emotional face flanker tasks, with the information load gradually increasing across schematic faces, same real faces, and different real faces. The results showed that the %Error increased in an orderly manner, and the flanker effect decreased in the different real-face tasks in the ASD group, further verifying the above hypothesis. Eriksen’s model (18) divided the cognitive processing into input end and output end. The influencing factor at the input end was the physical properties of the flanker stimuli, while the influencing factor at the output end was the congruency or incongruency of the preset response between flanker stimuli and target stimuli. From the former aspect, the intensifying physical attributes of flanker stimuli could promote sensitivity to processing target stimuli in children with ASD under a low information load (65). In contrast, as the information load increased, different face stimuli weakened the physical properties of flankers, thereby hindering the relatively optimized cognitive strategies of children with ASD. Consequently, we observed that children with ASD presented a higher %Error in both congruent and incongruent conditions compared to TD children in the different real-face task. Considering the output end, Murray et al. (66) and Wang et al. (67) suggested that individuals’ consumption of cognitive resources is limited under a low task load, and the remaining resources are then used to process interference stimuli automatically. Based on this, we inferred that the complexity of flankers in the different real-face tasks hampered children with ASD from extracting symbolic clues for further response, causing the flanker effect to diminish until it disappeared. Notably, we also found that the RT was negatively correlated with the %Error in the different real-face task in the ASD group. It is known that impulsive reactions are faster than cautious responses (44). Reduced selective attention to social clues may bias individuals with ASD toward more rapid decision-making; however, the loss of important information could increase response errors (46). Thus, we inferred that under a low information load, children with ASD were able to achieve enough response accuracy compared to TD children, albeit at the cost of spending more time. However, when the social information load increased and their cognitive resources were exhausted, they became gradually incompetent and might have impulsive responses.

Overall, our study elucidated that children with ASD behaved inefficiently in social–emotional interference control and faced more difficulties as the information load increased, which might affect their ability to adapt to and engage in social situations. More importantly, instead of showing completely impaired interference control on social–emotional information, children with ASD employed their unique but fragile cognitive strategies (rule-based strategies) (32). On the one hand, we should not only realize the disorder itself but also respect the differences rooted in autistic traits (68). Children with ASD face difficulties in social–emotional interference control and require support to optimize their cognitive strategies. One misconception in intervention is providing too much information all at once without adequate support, which can result in sensory overload and hinder children with ASD from acquiring new skills (69). On the other hand, previous studies have indicated that excessive reliance on rule-based strategies can make it more challenging for children with ASD to develop sufficiently fine-grained prototypes (25, 32, 54, 70, 71). Another common misconception in intervention is that extreme efforts are used to teach children with ASD to recognize single basic facial emotions. However, the observed improvements may be due to their well-practiced cognitive strategies rather than an enhancement in emotional perception. As a result, children with ASD are still prone to collapsing in complex environments, especially in conflict contexts. Therefore, targeted interventions to enhance their ability to deal with complex social scenarios could be achieved through a gradual transition from virtual emotional faces to real faces (72, 73), and from single faces to multiple faces and different faces (33, 74).

## 5 Limitations and prospects

While our work expands the understanding of interference control regarding social–emotional information in school-aged children with ASD, several limitations should be considered. First, the generalizability of our findings may be limited as we only included participants with IQs above 80. Given that nearly a third of children with ASD also have intellectual disability (2), and response times in flanker tasks are correlated with IQ among children with ASD (see **Supplementary Table SR3**), further research is required to account for the factor of intelligence, which may enhance the understanding of these findings. Second, we recruited a group of subjects spanning a wide age range, from childhood to adolescence. In our study, the response times in flanker tasks were correlated with age among children with ASD (see **Supplementary Table SR3**). Some previous studies, but not all, indicated that for individuals with ASD, the observed impairments of interference control during childhood might be partially resolved, or compensatory strategies might be developed in early adolescence (9, 75). Longitudinal studies that account for intraindividual covariation will be important for elucidating this effect. Third, although our study introduced schematic face stimuli as a transition between nonsocial and social information to analyze individuals’ cognitive strategies for social–emotional interference control at the behavioral level, the potential paths remain indirect. Eye-tracking and functional magnetic resonance imaging (fMRI) techniques can be applied in future studies to depict visual trajectories and deepen our understanding of the underlying mechanisms.

## 6 Conclusion

The current study supports the notion of inefficient interference control for social–emotional information in school-aged children with ASD. Specifically, we observed that children with ASD presented different cognitive strategies when confronting emotional face interference compared to TD children, indicating that they may adopt relatively optimized but limited compensatory strategies like symbolization. These cognitive strategies in the ASD group may be further challenged along with the increase in social information load. Our findings contribute to understanding the profile of social–emotional interference control in children with ASD and offer constructive insights in developing targeted behavioral interventions.

## Data Availability

The raw data supporting the conclusions of this article will be made available by the authors, without undue reservation.
