# Supplementary material for: Inefficient and unique processing of social–emotional interference in school-aged children with high-functioning autism spectrum disorder
Source: Front Psychiatry. 2024 Oct 9;15:1412533. doi: 10.3389/fpsyt.2024.1412533 (PMC11539409; doi:10.3389/fpsyt.2024.1412533)
Supplement: Supplementary file 1 [file Table1.docx]

**Table S1 Group differences in RT** **controlling for children’s age and full-scale intelligence quotient**

| **Stimulus Types** | **Conflict Condition** | **ASD（n=53）**  **Mean** ±**SD** | **TD（n=53）**  **Mean** ±**SD** | ***F*** | ***P*** ^a^ |
| --- | --- | --- | --- | --- | --- |
| **Arrow** | **Congruent Condition** | 950.17±334.29 | 704.67±174.77 | 19.302 | **<0.001** |
|  | **Incongruent Condition** | 1078.07±386.26 | 799.90±257.21 | 15.192 | **<0.001** |
| **Schematic Face** | **Congruent Condition** | 1002.65±211.41 | 873.75±192.73 | 6.550 | **0.012** |
|  | **Incongruent Condition** | 1090.29±259.88 | 905.96±207.75 | 11.361 | **0.001** |
| **Same Real Face** | **Congruent Condition** | 1192.85±255.94 | 1106.95±216.79 | 1.054 | 0.307 |
|  | **Incongruent Condition** | 1275.63±298.03 | 1174.01±227.52 | 1.943 | 0.166 |
| **Different Real Face** | **Congruent Condition** | 1240.13±329.96 | 1148.36±238.40 | 1.072 | 0.303 |
|  | **Incongruent Condition** | 1205.92±313.29 | 1161.58±251.95 | 0.025 | 0.875 |

Note: The values marked in bold means *p* < 0.05.

Abbreviations: RT, response time; ASD, autism spectrum disorder; TD, typically developing; SD, standard deviation.

^a^ The *p*-value for analysis of covariance between two groups controlling for children’s age and full-scale intelligence quotient.

**Table R1 Test for normality of RTs and** **%Errors in ASD and TD groups**

|  | **Stimulus Types** | **ASD(n=53)** | |  | **TD(n=53)** | |
| --- | --- | --- | --- | --- | --- | --- |
|  |  | ***Z*** | ***P*** |  | ***Z*** | ***P*** |
| **RT(ms)** | **Arrow** | 0.122 | 0.047 |  | 0.125 | 0.039 |
|  | **Schematic Face** | 0.061 | 0.200 |  | 0.084 | 0.200 |
|  | **Same Real Face** | 0.104 | 0.200 |  | 0.074 | 0.200 |
|  | **Different Real Face** | 0.081 | 0.200 |  | 0.076 | 0.200 |
| **%Error(%)** | **Arrow** | 0.326 | <0.001 |  | 0.289 | <0.001 |
|  | **Schematic Face** | 0.217 | <0.001 |  | 0.153 | 0.003 |
|  | **Same Real Face** | 0.150 | 0.005 |  | 0.216 | <0.001 |
|  | **Different Real Face** | 0.116 | 0.072 |  | 0.160 | 0.002 |

Note:

Abbreviations: ASD, autism spectrum disorder; TD, typically developing; RT, response time; %Error, percentage of error.

The *p*-value for Kolmogorov-Smirnov test.

**Table R2 The partial correlation analysis of the %Errors (and Δ%Errors) and SRS scores controlling for children’s age and full-scale intelligence quotient in ASD group**

| **Stimulus Types** | **Metrics** | **SRS total score** | **Social awareness** | **Social cognition** | **Social communication** | **Social motivation** | **Restricted and repetitive behaviors** |
| --- | --- | --- | --- | --- | --- | --- | --- |
| **Arrow** | **%Error ^Con^** | 0.199 | 0.175 | 0.185 | 0.201 | 0.169 | 0.050 |
|  | **%Error ^Inc^** | 0.115 | 0.061 | 0.172 | 0.129 | 0.041 | 0.009 |
| **Schematic Face** | **%Error ^Con^** | -0.192 | -0.009 | **-0.293** | -0.204 | -0.121 | -0.112 |
|  | **%Error ^Inc^** | 0.188 | 0.099 | 0.181 | 0.190 | -0.026 | 0.232 |
| **Same Real Face** | **%Error ^Con^** | -0.045 | 0.108 | -0.030 | -0.019 | -0.214 | -0.024 |
|  | **%Error ^Inc^** | 0.264 | 0.175 | **0.324** | 0.248 | 0.003 | 0.240 |
| **Different Real Face** | **%Error ^Con^** | -0.188 | -0.029 | -0.198 | -0.119 | -0.258 | -0.162 |
|  | **%Error ^Inc^** | 0.033 | 0.160 | 0.060 | 0.055 | -0.121 | -0.004 |
| **Arrow** | **Δ%Error** | 0.049 | -0.001 | 0.115 | 0.063 | -0.019 | -0.009 |
| **Schematic Face** | **Δ%Error** | **0.302** | 0.109 | **0.350** | **0.311** | 0.039 | **0.305** |
| **Same Real Face** | **Δ%Error** | **0.356** | 0.115 | **0.414** | **0.315** | 0.190 | **0.308** |
| **Different Real Face** | **Δ%Error** | 0.253 | 0.241 | **0.298** | 0.204 | 0.131 | 0.177 |

Note: The values marked in bold means *p* < 0.05.

Abbreviations: %Error ^Con^, percentage of error in congruent condition; %Error ^Inc^, percentage of error in incongruent condition, Δ%Errors, flanker effect of %Error.

**Table R3 The Spearman correlation analysis of the (RTs and %Errors) and (IQ and age) in the two groups**

| **Stimulus Types** | **Metrics** | **Age** | |  | **FSIQ** | |
| --- | --- | --- | --- | --- | --- | --- |
|  |  | **ASD** | **TD** |  | **ASD** | **TD** |
| **Arrow** | **RT ^Con^** | **-0.604** | **-0.385** |  | **-0.372** | -0.138 |
|  | **RT ^Inc^** | **-0.571** | **-.0380** |  | **-0.326** | -0.144 |
| **Schematic Face** | **RT ^Con^** | **-0.351** | -0.249 |  | **-0.300** | -0.117 |
|  | **RT ^Inc^** | **-0.338** | **-0.309** |  | **-0.383** | -0.033 |
| **Same Real Face** | **RT ^Con^** | **-0.498** | **-0.436** |  | **-0.312** | 0.117 |
|  | **RT ^Inc^** | **-0.446** | **-0.337** |  | -0.062 | 0.023 |
| **Different Real Face** | **RT ^Con^** | **-0.446** | **-0.372** |  | -0.098 | 0.019 |
|  | **RT ^Inc^** | **-0.468** | **-0.422** |  | -0.103 | 0.094 |
| **Arrow** | **%Error ^Con^** | -0.172 | 0.004 |  | -0.100 | **-0.293** |
|  | **%Error ^Inc^** | -0.150 | 0.106 |  | -0.015 | -0.152 |
| **Schematic Face** | **%Error ^Con^** | 0.142 | 0.172 |  | -0.260 | -0.127 |
|  | **%Error ^Inc^** | 0.189 | 0.043 |  | 0.013 | -0.180 |
| **Same Real Face** | **%Error ^Con^** | 0.213 | 0.122 |  | -0.144 | 0.057 |
|  | **%Error ^Inc^** | -0.052 | 0.209 |  | -0.122 | -0.128 |
| **Different Real Face** | **%Error ^Con^** | 0.093 | 0.208 |  | -0.244 | **-0.327** |
|  | **%Error ^Inc^** | 0.038 | 0.155 |  | -0.162 | -0.249 |

Note: The values marked in bold means *p* < 0.05.

Abbreviations: RT ^Con^, response time in congruent condition; RT ^Inc^, response time in incongruent condition; %Error ^Con^, percentage of error in congruent condition; %Error ^Inc^, percentage of error in incongruent condition; ASD, autism spectrum disorder; TD, typically developing.
